# Hormetic Effect of Berberine Attenuates the Anticancer Activity of Chemotherapeutic Agents

**DOI:** 10.1371/journal.pone.0139298

**Published:** 2015-09-30

**Authors:** Jiaolin Bao, Borong Huang, Lidi Zou, Shenghui Chen, Chao Zhang, Yulin Zhang, Meiwan Chen, Jian-Bo Wan, Huanxing Su, Yitao Wang, Chengwei He

**Affiliations:** 1 State Key Laboratory of Quality Research in Chinese Medicine, Institute of Chinese Medical Sciences, University of Macau, Taipa, Macao, China; 2 Institute of Chinese Materia Medica, China Academy of Chinese Medical Sciences, Beijing, China; Duke University Medical Center, UNITED STATES

## Abstract

Hormesis is a phenomenon of biphasic dose response characterized by exhibiting stimulatory or beneficial effects at low doses and inhibitory or toxic effects at high doses. Increasing numbers of chemicals of various types have been shown to induce apparent hormetic effect on cancer cells. However, the underlying significance and mechanisms remain to be elucidated. Berberine, one of the major active components of *Rhizoma coptidis*, has been manifested with notable anticancer activities. This study aims to investigate the hormetic effect of berberine and its influence on the anticancer activities of chemotherapeutic agents. Our results demonstrated that berberine at low dose range (1.25 ~ 5 μM) promoted cell proliferation to 112% ~170% of the untreated control in various cancer cells, while berberine at high dose rage (10 ~ 80 μM) inhibited cell proliferation. Further, we observed that co-treatment with low dose berberine could significantly attenuate the anticancer activity of chemotherapeutic agents, including fluorouracil (5-FU), camptothecin (CPT), and paclitaxel (TAX). The hormetic effect and thereby the attenuated anticancer activity of chemotherapeutic drugs by berberine may attributable to the activated protective stress response in cancer cells triggered by berberine, as evidenced by up-regulated MAPK/ERK1/2 and PI3K/AKT signaling pathways. These results provided important information to understand the potential side effects of hormesis, and suggested cautious application of natural compounds and relevant herbs in adjuvant treatment of cancer.

## Introduction

Hormesis is defined as a process in which exposure to a low dose of a chemical agent or environmental factor that is damaging at higher doses induces an adaptive beneficial effect on the cell or organism [1, 2]. In biology field, hormetic effect of cells or organisms can be considered an adaptive response to a moderate stress induced by physical, chemical, or biological factors [2]. Hormetic effect has been widely observed for a long time, but received increasing attention in recent years. Accumulating evidence suggests that hormesis, which mediated by low dose toxin or radiation treatment [3, 4], mild heat stress [5] or moderate regular exercise [6], show beneficial action to enhance body immunity and resistant to injury or diseases. In this respect, hormetic effect reveals beneficial implications on disease prevention and treatment. However, hormesis may also exhibit a potential adverse impact on cancer treatment with chemotherapeutic agents, which can often induce oxidative stress in cancer cells. Therefore, low dose cytotoxic agents may induce hormetic effect to stimulate cancer cell proliferation and tumor growth [7]. During the past 30 years, more than 120 chemical agents have shown hormetic effect on cancer cells from over 30 tissue types [7]. Moreover, low dose chemotherapeutic agents, such as bleomycin, chlorambucil, cisplatin, 5-fluorouracil, and doxorubicin, enhanced the proliferation of drug-sensitive or multidrug resistant cancer cells of various types [8–11]. In addition to chemotherapeutic agents, more often it was found that hormesis was induced by natural compounds isolated from plants [12], such as resveratrol, epigallocatechin, quercetin, genistein and flavonoids. In conventional pharmacological studies on anticancer agents, researchers mostly emphasize the anticancer efficacy of high dose agents, while overlook that low dose agents may stimulate cancer cell proliferation owing to the fact that the threshold dose response model is regarded as dominant dose-response model. However, studies indicated that more often the cytotoxic agents exhibited hormetic dose response model than threshold model in toxicological and pharmacological studies [13]. Thus, hormetic stimulatory effects of cytotoxic agents at low doses should be taken into account in the investigation of anticancer agents.

*Rhizoma coptidis* is a highly valued traditional Chinese medicine which has been widely applied in complementary and alternative medicines in China, Korea, India, Japan, and other Asian countries, especially for the treatment of dysentery, cancer, diabetes mellitus, and eczema, mostly used in formulas [14]. Berberine is one of the major active components of *Rhizoma coptidis*, which is an isoquinoline alkaloid with abundant pharmacological activities [15]. Berberine hydrochloride has been approved by China Food and Drug Administration as anti-dysentery drug for many years. In recent years, berberine has been demonstrated significant anticancer activities in various types of cancer [16, 17]. Moreover, berberine also showed synergistic anticancer effects in combination treatment of cancer with chemotherapeutic agents [18, 19] or radiotherapy [20]. The encouraging results of studies suggest that berberine might have potential to be developed as an effective adjuvant anticancer agent. However, the hormetic dose response of berberine has not been evaluated yet. This information could be imperative to provide suggestions for potential clinical applications, particularly in the treatment of cancer.

In this study, we investigated whether berberine at relative low doses stimulates the growth of cancer cells, i.e. hormetic effect, and whether this effect attenuates the anticancer activities of chemotherapeutic agents. The underlying molecular mechanisms of hormesis induced by berberine were also investigated.

## Materials and Methods

### Reagents

Berberine hydrochloride (BER), 5-Fluorouracil (5-FU) camptothecin (CPT), and Paclitaxel (TAX) were purchased from Sigma-Aldrich (St. Louis, MO, USA). WST-1 cell proliferation and cytotoxicity assay kit, PD98059, and LY294002 were purchased from Beyotime (Haimen, Jiangsu, China). RPMI 1640 medium, Dulbecco's modified Eagle's medium (DMEM), penicillin-streptomycin, trypsin, fetal bovine serum (FBS), and phosphate buffered saline (PBS) were obtained from Gibco (Carlsbad, CA, USA). Antibodies against phosphor-ERK, ERK, phosphor-AKT, AKT and β-actin were purchased from Cell Signaling Technology, Inc. (Beverly, MA, USA).

### Cell culture

B16-F10 murine melanoma cell line was purchased from Cell Bank of Type Culture Collection of Chinese Academy of Sciences (Shanghai, China). Human breast cancer cell lines MDA-MB-231, MDA-MB-468, and MCF-7, and human colon cancer cell line LS-174 cells were obtained from American Type Culture Collection (ATCC, Manassas, VA, USA). According to ATCC guideline, cells were cultured in basic medium supplemented with 10% heat-inactivated fetal bovine serum, 100 U/mL penicillin, and 100 μg/mL streptomycin at 37°C in a humidified atmosphere of 5% CO_2_. The medium was changed every other day.

### WST-1 assay

Cell proliferation was determined by WST-1 colorimetric assay [21]. Cancer cells were plated at a density of 2~6×10^3^ cells per well in 96-well plate. After 16~20 h incubation, the cells were treated with a series of concentrations of berberine for 24, 48, or 72 h at 37°C. WST-1 reagent (10 μl) was added to each well at 2 h before the endpoint of incubation. The cells were further incubated for 2 h at 37°C, then the absorbance was measured by a SpectraMax® M5 microplate reader (Molecular devices, Sunnyvale, CA) with a test wavelength at 450 nm and a reference wavelength at 690 nm. Inhibitory rate of cell proliferation was calculated comparing with OD value of control group. All assays were performed at least three replicates and repeated three times.

### Western blotting

After treatment, cells were washed twice with PBS and lysed with RIPA lysis buffer containing 1% phenylmethylsulfonyl fluoride (PMSF) and 1% protease inhibitor cocktail (Thermo, Rockford, IL, USA). The concentration of protein samples was determined using a BCA protein assay kit (Thermo, Rockford, IL, USA). Equivalent amounts of proteins from each group were separated using SDS-PAGE electrophoresis, followed by transferring onto an immun-blot PVDF membrane (Bio-Rad, Philadelphia, PA, USA). After being blocked for 1 h in 5% non-fat dried milk with PBST buffer, the membrane was incubated with 1:1000 dilutions of primary antibody at 4°C overnight. β-actin was used as the internal control. PVDF membranes were washed three times in PBST buffer, and followed by incubation with 1: 5000 dilutions of the corresponding second antibody. Specific protein bands were visualized using an ECL advanced Western blotting detection kit (GE Healthcare, Buckinghamshire, UK). The density of the bands was quantified by Quantity One Software.

### Statistical analysis

Data were expressed as the mean ± standard deviation. One-way ANOVA and Turkey’s Multiple Comparison Test are used in the GraphPad Prism 5.0 software (GraphPad Software, Inc., San Diego, CA). The value of statistical significance is set at *p* < 0.05.

## Results

### BER induced hormetic effect in various cancer cell lines

**Fig 1:**
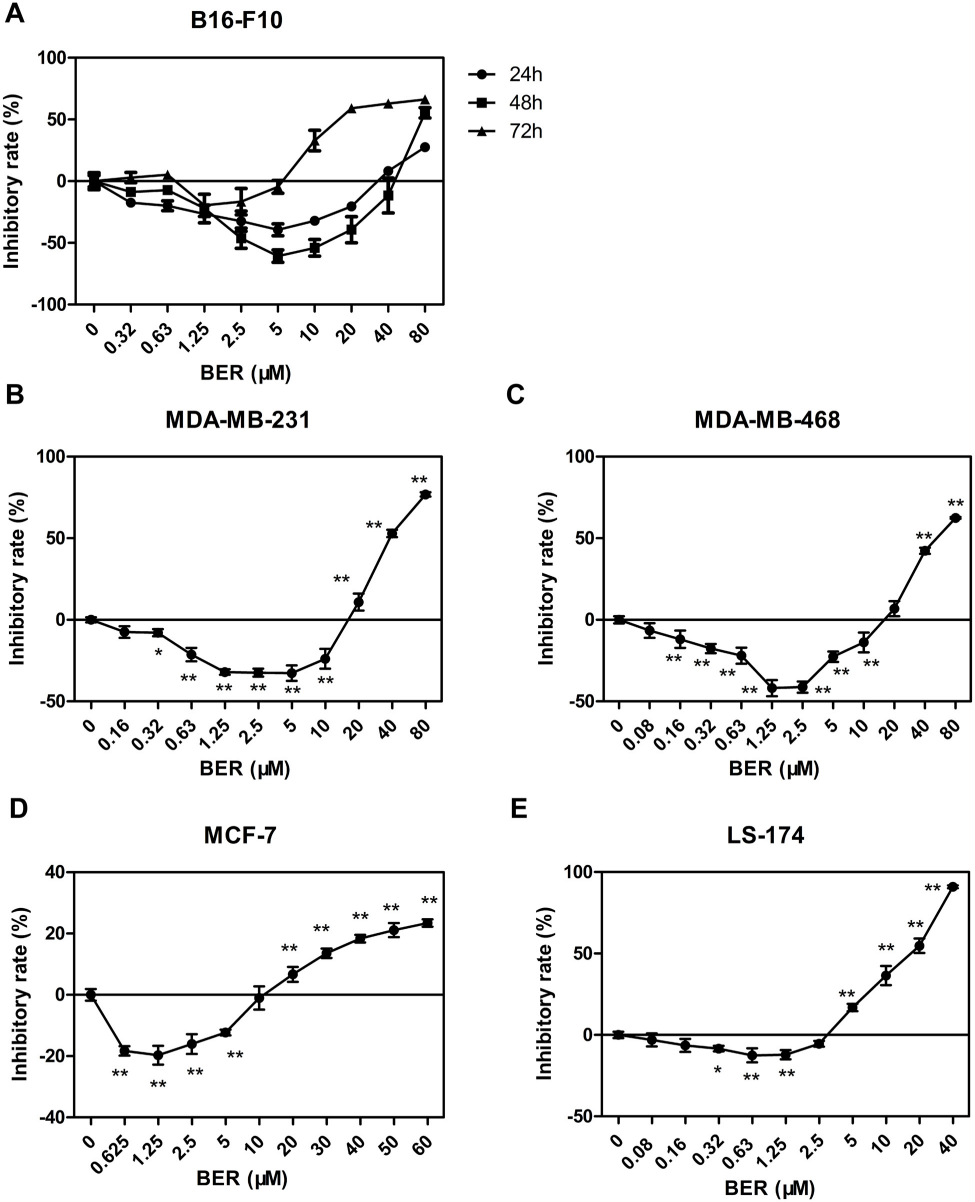
BER induced hormetic effects on various cancer cell lines. B16-F10 cells were treated with various concentrations of BER for 24, 48, or 72 h, respectively. Other cells (MDA-MB-231, MDA-MB-468, MCF-7, and LS-174) were treated with various concentrations of BER for 72 h. Cell viability was determined by WST-1 colorimetric assay. Inhibitory rate was calculated comparing to OD value of untreated control group. Data were expressed as means ± SD (*n* ≥ 3). * *P* < 0.05, ** *P* < 0.01, compared to untreated control.

To investigate the hormetic effect of berberine (BER), we tested five cancer cell lines, murine melanoma cell line B16-F10, human breast cancer cell line MDA-MB-231, MDA-MB-468 and MCF-7, and human colon cancer cell line LS-174. Cell viability was determined by WST-1 colorimetric assay. We observed the temporal features of hormetic effect induced by BER on B16-F10 cells. BER at relatively low concentrations significantly stimulated cell growth, while high concentration of BER inhibited cell growth of the tested cancer cell lines. This biphasic dose-response phenomenon was consistent with the typical feature of hormesis [22]. The result showed that treatment of BER for 48 h exhibited the greatest growth stimulation on B16-F10 cells with a maximum stimulatory rate of about 70% when BER was at 5 μM, comparing to the groups of treatment for 24 h and 72 h (Fig 1A). As shown in Fig 1B to 1E, the hormetic effects of BER were shown in all tested cancer cells after 72 h treatment. However, the maximum stimulatory effects on cell growth and the corresponding range of doses of BER varied in different cancer cell types. BER (1.25μM) maximally stimulated the growth of breast cancer cells (MDA-MB-231 and MDA-MB-468) by 40%, while BER of the same dose only stimulated the growth of colon cancer LS-174 cells by 12% maximally. These results demonstrated that BER exhibited a typical hormetic dose response in cancer cells.

### Hormetic effect of BER attenuated the in vitro anticancer activity of CPT, TAX and 5-FU in B16-F10 melanoma cells

**Fig 2:**
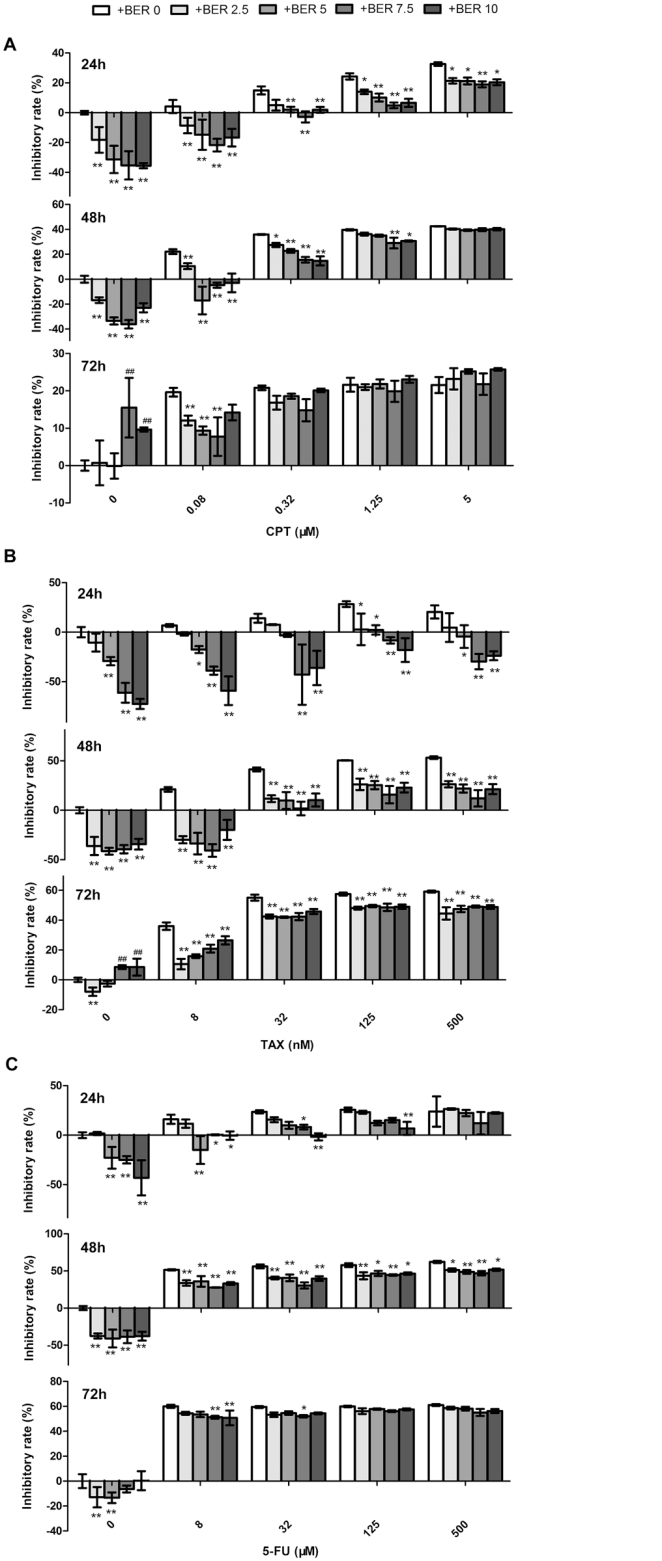
Low doses of BER attenuated the in vitro anticancer activity of chemotherapeutic agents (CTA) in B16-F10 cells. B16-F10 cells were co-treated with different concentrations of CPT (A), TAX (B), or 5-FU (C) and BER for 24 h, 48 h and 72 h, respectively, before the determination of cell viability by WST-1 assay. Inhibitory rate was calculated comparing to OD value of untreated control group. Data are expressed as mean ± SD (*n* ≥ 3). * *P* < 0.05, ** *P* < 0.01, compared to the group of CTA used alone.

We hypothesized that the hormetic effect of BER could attenuate the anticancer activities of chemotherapeutic agents. To test this hypothesis, low concentrations of BER (2.5–10 μM) combined with camptothecin (CPT), paclitaxel (TAX) or 5 fluorouracil (5-FU) were used to treat B16-F10 cells for 24 h, 48 h and 72 h. As shown in Fig 2A, CPT inhibited the growth of B16-F10 cells in a dose- and time-dependent manner. Co-treatment of low doses of BER (BER) significantly attenuated the growth inhibition of CPT against B16-F10 cells, for example, the inhibitory rate of 24 h was decreased from 24.3% to 4.9% in the group of CPT (1.25 μM) plus BER (7.5 μM) comparing to CPT used alone. Similar results were observed in a longer time treatment. The growth inhibition of CPT at 0.08 μM was even completely reversed by BER when co-treatment was applied for 24 h and 48 h. Similar results were observed in the combined treatment of low doses of BER with TAX or 5-FU (Fig 2B and 2C). The in vitro anticancer activity of TAX was greatest inhibited by low doses of BER. Particularly, BER reversed the growth inhibition of TAX at all tested concentrations for 24 h treatment. However, the attenuation of anticancer activity of chemotherapeutic agents by low doses of BER was greatly decreased or lost when the co-treatment extended to 48 h or 72 h. These results demonstrated that the hormetic effects of BER significantly attenuated the in vitro anticancer activity of tested chemotherapeutic agents. The degree of attenuation was dependent on the duration of treatment, the concentration and type of anticancer agents. The data provided important information to discern the biomedical significance of hormesis, and suggested a cautious application of BER both used alone or in combination with other agents in cancer treatment.

### Low dose BER up-regulated the MAPK/ERK and PI3K/AKT pathways

**Fig 3:**
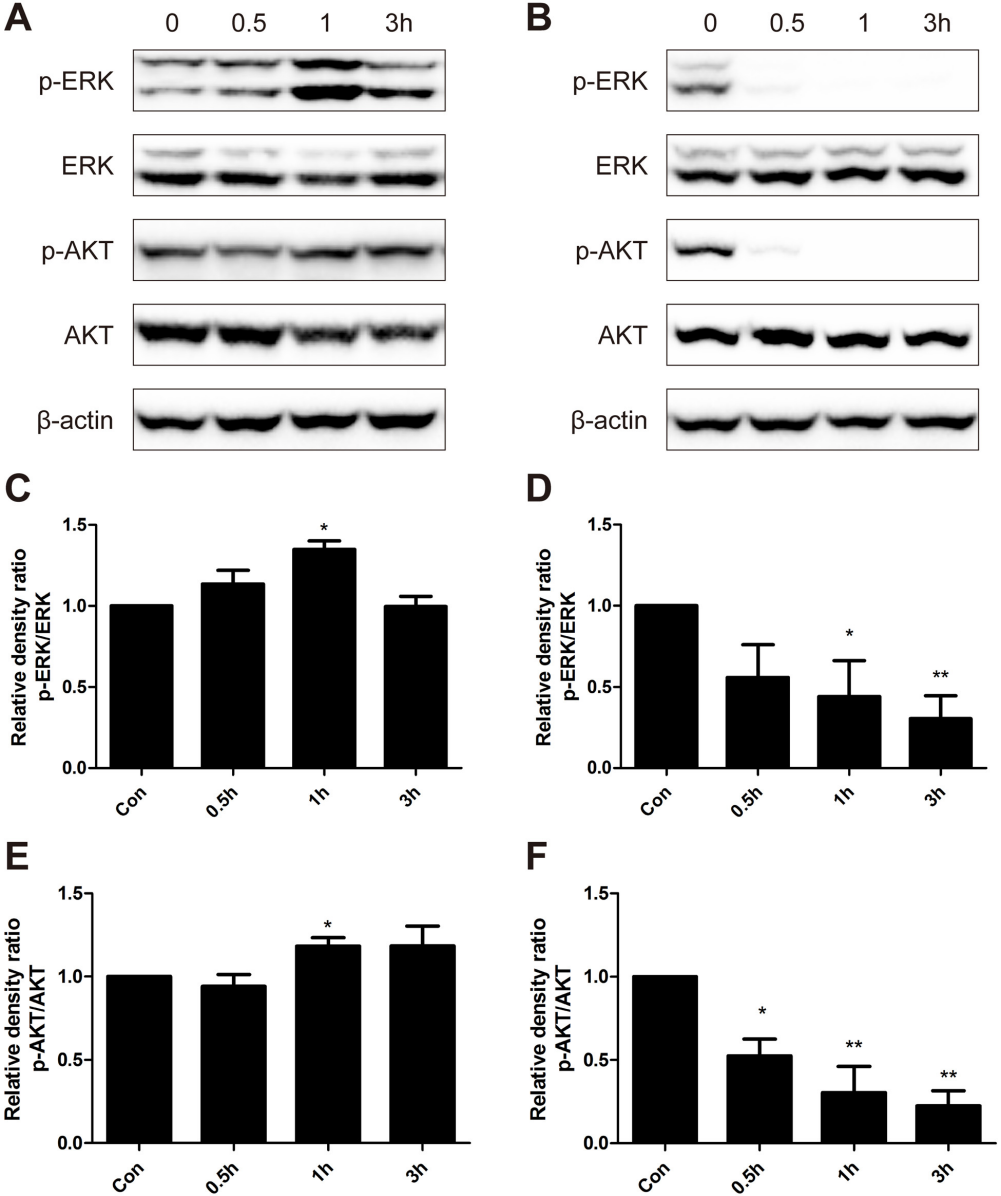
Western blot analysis of protein expression levels of p-ERK, ERK, p-AKT and AKT. B16-F10 cells were treated with low dose of BER (5 μM) (A) and high dose of BER (40 μM) (B) for 0.5 h, 1 h and 3 h, respectively. Total protein of cell lysates was extracted and subjected to Western blot analysis. The density of each band was quantified by Quantity One Software, and the relative density ratio of each protein was calculated accordingly. β-actin was used as the internal control. Data are expressed as mean ± SD (*n* ≥ 3). * *P* < 0.05, ** *P* < 0.01 compared to untreated control.

MAPK/ERK1/2 and PI3K/AKT pathways play an important role in cell proliferation and survival [23, 24], and adaptive oxidative response [25, 26]. We hypothesized that MAPK/ERK and PI3K/AKT signaling pathways were involved in the hormetic effect induced by low dose BER. Hence, we examined the phosphorylated and total protein levels of ERK1/2 and AKT in B16-F10 cells treated with low (5 μM) and high doses (40 μM) of BER. Representative results from each group were presented in Fig 3. Density analysis showed that ratios of phosphorylated ERK to total ERK were significantly increased with a peak increase of about 40% after treatment with 5 μM BER for 1 h (Fig 3A). However, the expression of phosphorylated ERK in B16-F10 cells decreased sharply over time after treatment with high dose of BER (40 μM) (Fig 3B). The relative density ratio of phosphorylated AKT to total AKT was also increased after treatment of 5 μM BER, and had a rapid decline when treated with high dose BER. These results indicated that low dose BER activated MAPK/ERK and PI3K/AKT signaling pathways, which might be responsible for the hormetic effect and inhibition of anticancer activity of chemotherapeutic agents by BER.

### The hormetic effect of BER was reversed by inhibition of MAPK/ERK and PI3K/AKT signaling pathways

**Fig 4:**
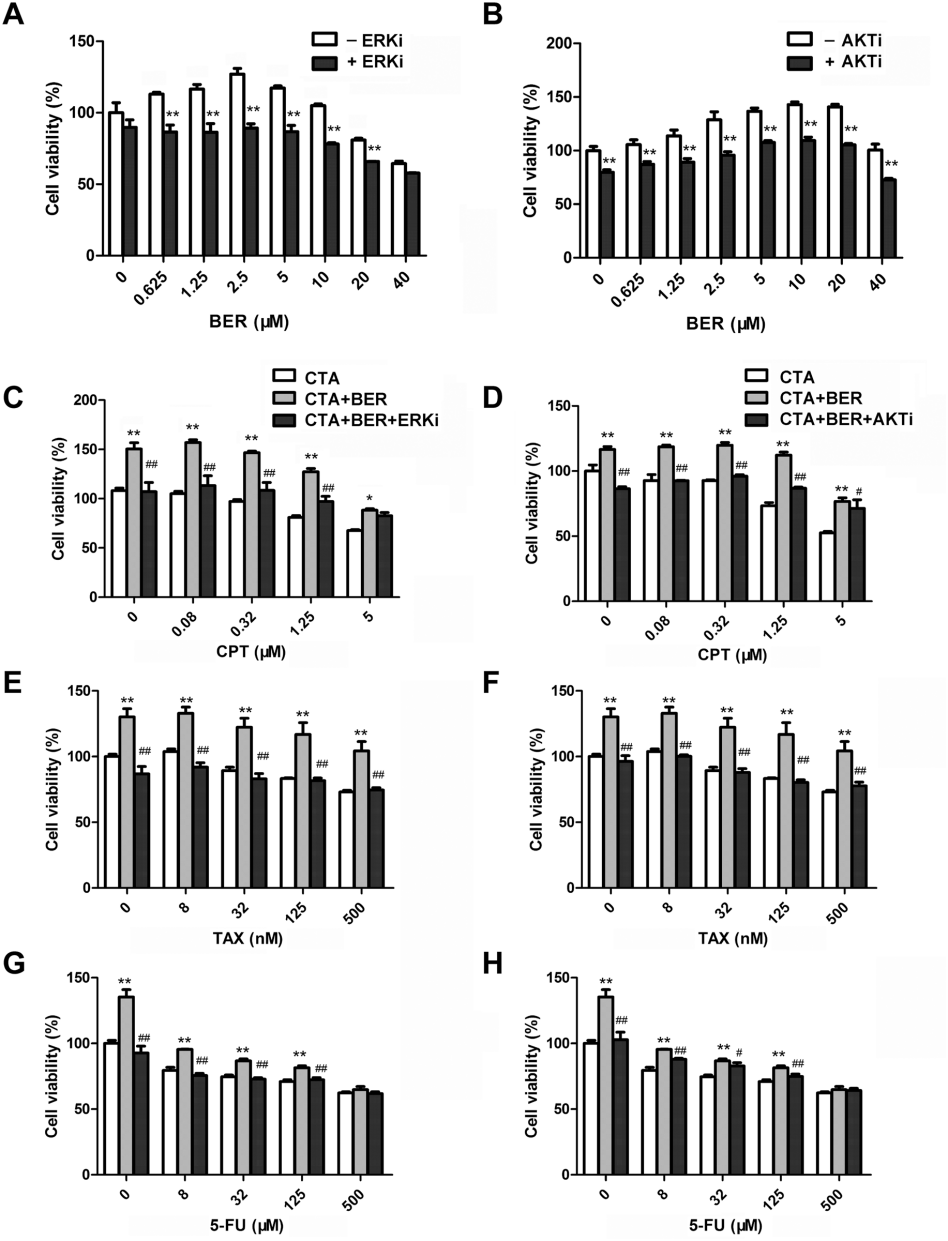
ERK and AKT inhibitors reversed the hormetic effect of BER and restored the in vitro anticancer activity of chemotherapeutic agents (CTA). B16-F10 cells were pretreated with 20 μM ERK inhibitor (ERKi) PD98059 (A, C, E, G) or 5 μM AKT inhibitor (AKTi) LY294002 (B, D, F, H) for 1 h. Cell viability was determined by WST-1 assay after 24 h treatment of 5 μM BER alone (A, B) or combined with CTA (C-H). Data were expressed as mean ± SD (*n* ≥ 3). *P < 0.05, **P < 0.01 compared to BER (A, B) or CTA (C-H) used alone. ^#^ *P* < 0.05, ^##^ *P* < 0.01 compared to combination treatment of CTA and low dose BER.

To further validate the role of ERK and AKT signaling pathways in hormetic dose response of BER, we tested whether the pathway inhibitors could affect BER-induced cell growth stimulation in B16-F10 cells. As shown in Fig 4A and 4B, cell growth of B16-F10 cells was increased after treatment with low dose BER, which was consistent with previous results (Fig 1A). In the presence of 20 μM MAPK/ERK kinase (MEK) inhibitor PB98059, the growth stimulation by low dose BER was completely abolished (Fig 4A). Similarly, PI3K inhibitor LY294002 partially abolished the growth stimulation by low dose BER (Fig 4B). These results demonstrated that MAPK/ERK and PI3K/AKT pathways were involved, at least partially, in the hormetic effect of BER on B16-F10 cells.

### MEK and PI3K inhibitors restored the anticancer activity of chemotherapeutic agents

To investigate whether ERK and AKT activation participates in the attenuation of anticancer activity of chemotherapeutic agents by low dose BER, B16-F10 cells were exposed to chemotherapeutic agents alone, or in combination with low dose BER (5 μM) and MEK or PI3K inhibitors. As shown in Fig 4C–4H, 5 μM BER significantly stimulated the growth of B16-F10 cells, and attenuated the in vitro anticancer activity of tested chemotherapeutic agents. In the presence of 20 μM MEK inhibitor PD98059, the growth inhibition of CPT, TAX, and 5-FU was almost completely restored comparing to they were used alone or in combination with BER (Fig 4C, 4E and 4G). Similarly, 5 μM PI3K inhibitor LY294002 restored the in vitro anticancer activity of CPT, TAX, and 5-FU (Fig 4D, 4F and 4H). These results demonstrated that MAPK/ERK and PI3K/AKT pathways were involved, at least partially, in the attenuation of in vitro anticancer activity of the tested chemotherapeutic agents by low dose BER in B16-F10 cells.

## Discussion

Berberine, one of the major active ingredients in *Rhizoma coptidis*, a widely used traditional Chinese medicine, has a wide range of pharmacological activities [14, 27]. In recent years, a growing number of studies had reported the anticancer activity of berberine in various types of cancers, either used alone and in combination with chemotherapeutic agents [18, 19], suggesting its potential in developing as a promising adjuvant anticancer drug. On the other hand, previous studies demonstrated that more than 120 chemical agents, particularly natural compounds, exhibited hormetic effects on cancer cells, i.e. stimulated cancer cell growth at low doses [7, 12]. More strikingly, chemotherapeutic agents at low doses promoted the proliferation of cancer cells of different types [8–11]. These findings imply more concerns of safety and side effects of chemotherapeutic agents as well as natural compounds due to hormetic low-dose stimulation to cancer cells. It is inevitably that there is a low dose window of a drug in the organism because of pharmacokinetics (drug absorption, distribution, metabolism, and excretion). Therefore, it is crucial to determine whether a potential anticancer agent exhibits hormetic effects on cancer cells, as well as the stimulatory strength and the range of low doses. In the present study, we firstly demonstrated that the effects of berberine on cell growth exhibited typical hormetic dose response in several cancer cell lines, i.e. relative low doses of berberine stimulated the growth of cancer cells, while high doses of berberine inhibited cell growth. Moreover, low dose berberine remarkably attenuated the in vitro anticancer activity of chemotherapeutic agents. Our data further indicated that mitogen-activated protein kinases (MAPK)/ERK and phosphoinositide 3-kinase (PI3K)/AKT signaling pathways were activated by low dose berberine, and involved, at least partially, in the hormetic effect of berberine and its inhibition on the anticancer activity of chemotherapeutic drugs.

The characteristic of hormesis is a biphasic dose-response curve, with stimulatory or beneficial effect at low doses and inhibitory or toxic effect at high doses [1, 2]. The range of maximum stimulatory response is about 30% to 60% above that of the control, and the width of the stimulatory dosage is mostly within 100-fold of the threshold value [4]. Our results showed that the maximum stimulatory rate was nearly 70% on B16-F10 cells after 48 h treatment of 5 μM berberine, and the range of the stimulatory dosage is about 120-fold below the threshold value (Fig 1A), indicating that the two phasic dose response of berberine is a typical hormetic phenomenon. Furthermore, we found that low dose berberine also enhanced the growth of other cancer cells, including breast cancer MDA-MB-231, MDA-MB-468, and MCF-7 cells, and colon cancer LS-174 cells (Fig 1B–1E). The degree of growth stimulation and the dosage range of berberine notably varied among different types of cancer cells. Our results shed light on the universal hormetic phenomenon induced by phytochemical compounds, such as resveratrol, curcumin, epigallocatechin, etc. [12].

In clinical practice, drug combination is a common-use strategy for cancer treatment, which may increase therapy sensitivity, provide multiple drug targets and avoid potential drug resistant. Berberine also showed synergistic anticancer activity combined with other chemotherapeutic drugs as reported previously [18, 19]. However, in our present study, in contrast to its synergistic anticancer activity at high doses, low dose berberine significantly reduced, or even reversed the anticancer activity of camptothecin (CPT), paclitaxel (TAX), and 5 fluorouracil (5-FU) in B16-F10 cells. As shown in Fig 2B, the anticancer activity of TAX was greatest inhibited by low doses of berberine. Comparing to groups of 48 h and 72 h, co-treatment of low dose berberine for 24 h exhibited the highest inhibition on the anticancer activity of chemotherapeutic agents (Fig 2), which is consistent with the time-dependent manner of hormetic effects induced by berberine (Fig 1). These results confirmed the speculation that hormetic effect of agents could interfere with the anticancer activity of chemotherapeutic drugs [4]. The degree of interference was related to the duration of treatment, the concentration and type of anticancer agents. Therefore, we should be aware of the hormetic dose response of berberine both used alone and in combination with other agents in cancer treatment. An appropriate treatment regime should be carefully arranged and blood concentration of berberine should be monitored in clinical applications to avoid potential unwanted side effects. More studies in this respect are required, and it is interesting to examine the hormetic dose response and the potential adverse effects of more natural compounds and existing anticancer drugs at low doses.

Hormesis was considered a universal phenomenon in many disciplines of biological and medical sciences, such as toxicology, immunology, aging biology, neuroscience, microbiology, radiology, etc. [1]. It has been reported that key signaling pathways of cell survival/ proliferation and oxidative stress response were involved in the processes of hormesis [12], including MAPK/ERK1/2 [28, 29], PI3K/AKT [30, 31], nuclear factor-erythroid 2p45 (NF-E2)-related factor (Nrf2)/antioxidant response element (ARE) [32, 33], etc. Demirovic et al [34] reported that curcumin induced a hormetic dose response in wound healing via oxidative stress response pathway Nrf2/ARE/HO-1. Pallas [35] and Mattson [36] summarized the mechanisms of hormesis induced by resveratrol, including activation of cell-survival signaling kinases, NRF2, cAMP response element-binding protein (CREB), components of sirtuin family, and AMP-activated protein kinase (AMPK). In our results (Fig 3), the ratios of phosphorylated ERK1/2 to total ERK1/2 were significantly increased with a peak increase of about 40% after treatment with berberine at 5 μM, and decreased rapidly when treated with high dose berberine at 40 μM, indicating that low dose berberine obviously activated both MAPK/ERK1/2 and PI3K/AKT signaling pathways. MAPK pathways play critical roles in the regulation of cell proliferation, differentiation, development, survival, apoptosis, and cellular stresses. Three subfamilies of MAPK have been well-characterized in vertebrate: ERK1/2, JNKs, and p38 MAPKs[37]. In MAPK/ERK1/2 signaling pathway, the activation of MAPKs can be provoked by tyrosine kinase receptors (RTKs), integrins and ion channels in multistep processes [38]. Extracellular cell growth factors or cytokines activate Raf via a variety of receptors, followed by the activation of MEK by the phosphorylated Raf, and the phosphorylation of ERK by the activated MEK. Finally, the activated ERK promotes cell proliferation and cell survival by regulating the activity of transcriptional factors and changing target gene expression [23]. The PI3K/AKT pathway also plays important roles in the regulation of cell growth, proliferation, survival, and apoptosis. The serine/threonine kinase Akt is a key mediator of the PI3K signaling pathway. PI3K catalyzes the formation of phosphatidylinositol (3,4,5)-trisphosphate (PIP3), which can stimulates the phosphorylation and activation of AKT. Then, the phosphorylated AKT promotes cell survival and proliferation through activating target transcriptional factors[39]. In this study, inhibitors of MEK (PB98059) and PI3K (LY294002) were applied to abolish the activation of ERK and AKT by low dose berberine. Consequently, the hormetic effect induced by berberine was suppressed and the anticancer activities of all three chemotherapeutic drugs were restored. These results demonstrated that MAPK/ERK1/2 and PI3K/AKT signaling pathways were involved in the hormetic effect and attenuation of anticancer drug activities by low dose berberine. However, the detailed mechanisms for their activation remain to be further investigated.

In summary, our study demonstrated that berberine induced a significant hormetic dose response, in which low dose berberine strongly stimulated the growth of cancer cells, in contrast to its anticancer activity at high doses. Moreover, low dose berberine greatly attenuated or even reversed the in vitro anticancer activity of chemotherapeutic drugs, including CPT, TAX, and 5-FU. These adverse effects were associated with the activation of signaling pathways for cell proliferation/survival and adaptive oxidative stress response in cancer cells, including MAPK/ERK1/2 and PI3K/AKT. As hormetic effect was considered a universal adaptive response to low dose (sub-lethal) cytotoxic agents, our study suggested cautious application of natural compounds and relevant herbs, which could cause obvious hormetic response, in adjuvant treatment of cancer.
